# Efficacy and Safety of Amiodarone and Propranolol in Pediatric Cardiology for Arrhythmia After Cardiac Surgery: A Retrospective Design Study

**DOI:** 10.7759/cureus.73102

**Published:** 2024-11-05

**Authors:** Amr Almomani, Mohammad A Obeidat, Mohammad H Khassawneh, Sakher M Maayeh, Khaled N Al-Malouf

**Affiliations:** 1 Pediatric Cardiology, Jordanian Royal Medical Services, Amman, JOR; 2 Internal Medicine, Jordanian Royal Medical Services, Amman, JOR; 3 Cardiac Surgery, Jordanian Royal Medical Services, Amman, JOR

**Keywords:** antiarrhythmic medication therapy, atrial septal defect closure, cardiac arrythmia, intravenous amiodarone, propranolol therapy, tricuspid valve repair

## Abstract

Objectives

The study evaluated the efficacy of antiarrhythmic pharmacotherapies in managing tachyarrhythmia episodes in pediatric patients with congenital heart diseases post-tricuspid valve repair, assessing reductions in haemodynamic parameters and symptomatic variables, and observing side effects.

Methods

From January 2020 to January 2024, this study reviewed data from 300 patients, aged up to 18 years, who experienced arrhythmia following cardiac surgery and received treatment with amiodarone, propranolol, or both. The information included demographic and anthropometric measures, haemodynamic parameters, and antiarrhythmic drugs used to treat arrhythmias before and after tricuspid valve repair. The study employed two validated symptomatic assessment scales: the four "A" symptomatic (4ASX) score and the New York Heart Association (NYHA) classification I-IV. We categorised the outcomes into two groups: the unresponsive cohort (Cohort I) and the responsive cohort (Cohort II). The study determined the antiarrhythmic efficacy by observing that the responsiveness group exhibited higher distribution rates in the lower heart rate category and higher decrementing rates in the tracked haemodynamics categories. The negative occurrence of at least one composited side effect of interest identified the safety significance profile for these three adopted antiarrhythmic interventions. The study adopted the threshold of 0.05 as the level of statistical significance.

Results

This study found that 32.33% exhibited clinical unresponsiveness to antiarrhythmic agents, while 67.67% achieved desired responsiveness. 38% used amiodarone as the choice antiarrhythmic medication, while 32.3% used propranolol. The study found significant distribution rates concerning heart rates, with 56.2% of patients with a heart rate of <100 bpm being responsive, compared to 38.1% in the unresponsive cohort. Symptomatic improvement was noted after transcatheter atrial septal defect closure in the 300 patients undergoing this procedure to address complications related to tricuspid regurgitation. Most of the adopted haemodynamic indices showed statistical significance decrementing rates in the responsiveness cohort compared to the unresponsiveness cohort.

Conclusion

Using antiarrhythmic pharmacotherapies during tricuspid valve repair had statistically significant responsiveness statuses against procedural arrhythmia, positive outcomes in symptomatic and haemodynamic monitored parameters, and a statistically significant noninferior safety profile regardless of the antiarrhythmic agent used (amiodarone, propranolol, or a combination of both.

## Introduction

Anatomic tricuspid valve (TV)* *abnormalities may be primary congenital anomalies or secondary in the context of other cardiac defects. Moreover, functional abnormalities of the TV may occur in various forms of congenital heart disease, affecting approximately 80% of patients with this condition. TV abnormalities are prevalent in congenital heart disease and may be inadequately identified if not actively investigated. TV imaging is an essential component of the evaluation for all patients with congenital heart disease (CHD) [1]. These valve abnormalities impair leaflet coaptation and can lead to regurgitation or stenosis. Tricuspid valve disease can result in atrial, nodal, and ventricular arrhythmias, which compromise functional status and worse outcomes [2]. Tricuspid valve abnormalities have a low predictive value on their own but contribute to increased risk when associated with other morphological features that create an enormous hemodynamic burden. The gold standard treatment is early curative surgery to normalize pulmonary and systemic hemodynamics [3]. Surgical techniques for tricuspid valve repair in CHD are available, including traditional options like tricuspid valve repair, bicuspidisation of the mixed tricuspid valve, and in situ or ex situ tricuspid valve replacement. However, elaborate on the innovative surgical options have been introduced in recent years [4]. Postoperative complications after isolated or combined TV repair can be severe, including pericardial effusion, re-operation, heart block, low cardiac output, severe reoperation, and atrioventricular block. Minimally invasive techniques have been shown to reduce surgical mortality, length of stay in the ICU, and total time of hospitalization, leading to more rapid recovery [5].

Arrhythmias are a common issue in patients with congenital heart disease, developing in many patients following tricuspid valve replacement or repair. Atrial arrhythmias, such as atrial flutter, can be short-lived or chronic, affecting symptoms such as heart flutters, palpitations, fatigue, and kidney function. Nodal or focal atrial arrhythmias may require further electrophysiological studies and ablation procedures to prevent further complications such as heart failure or sudden cardiac death [6]. Arrhythmias in congenital heart disease (CHD) patients are often identified through telemonitoring, Holter monitors, and electrophysiological studies. Common medications used to treat or manage arrhythmias include rhythm or rate control medications, anti-arrhythmic medications, systemic oral anticoagulation agents, and surgical or catheter-based ablation procedures. It is an important fact that propranolol, amiodarone, and flecainide are the most frequently utilised arrhythmia medications [7]. The medical management of rhythm versus rate control strategies in this population is impacted by age, concomitant heart issues, heart rhythm complexity, and prolonged duration of arrhythmia [8]. Management of arrhythmia in numerous congenital heart disease patients necessitates an interdisciplinary team comprising paediatric general and subspeciality cardiologists, paediatric and other professional electrophysiologists, nursing staff, pharmacists, social services and cardiac anaesthesiologists. Adherence is an important part of treating arrhythmias in CHD patients, and non-adherence is more common in adolescents and slightly older adult survivors and is associated with knowledge deficits for chronic medications [9].

The role of amiodarone and propranolol in treating arrhythmias in tricuspid valve repair patients is crucial, as they can reduce symptoms and functional valve class, and improve right ventricular size and function. Both drugs must be used while monitoring the patient for months or years due to their potential side effects [10]. Despite no randomized controlled trials supporting the preferred criteria in advanced therapy in the presence of ventricular dysfunction, there is growing evidence that both drugs are effective in controlling recurrent arrhythmic events. For these drugs used for chronic treatment, individualized optimization of the dose is needed, as well as close monitoring due to potential adverse drug-drug interactions. Future cohort studies about arrhythmic events post-repair are needed to investigate and validate a structured management plan for rhythm disorders following repair [11].

The study focused on the efficacy of the investigated antiarrhythmic pharmacotherapies in managing tachyarrhythmia episodes in paediatric patients with congenital heart diseases after tricuspid valve repair. It also assessed the corresponding reductions in haemodynamic parameters and symptomatic variables while observing for the positivity of antiarrhythmic major side effects during a period marked by a lack of evidence-based research on the effectiveness and safety of arrhythmia treatments in paediatric cardiology, aiming to enhance the existing body of knowledge.

## Materials and methods

This study conducted a retrospective review of the findings from patients admitted to the paediatric cardiology department at the Queen Alia Heart Institution, a tertiary referral centre for cardiology interventional specialities in our Royal Medical Services institutions, Amman, Jordan. The analysis included data from 300 patients aged up to 18 years who experienced arrhythmia following cardiac surgery and were treated with amiodarone, propranolol, or both, from January 2020 to January 2024. This study received preliminary approval from the Jordanian Royal Medical Services Institutional Review Board (JRMS_IRB) on August 13, 2024, under registration number 17_12/2024. This sanctioned study received official approval for publication following review by our institutional directorate of professional training and planning on October 8, 2024. This study meticulously adhered to the principles of the Helsinki Declaration.

Paediatric patients with retrievable data missing over 5% and those aged below 2 years or above 18 years were excluded from this study. The data was collected from patients admitted to the cardiology departments who subsequently developed arrhythmia during or after transcatheter cardiology interventions. This study examined specifically the transcatheter closure of atrial septal defects and tricuspid valve repair, to evaluate the efficacy of three tested antiarrhythmic drugs in patients with mild, moderate, and severe tricuspid regurgitation. 

The collected data included demographic and anthropometric indices such as gender and BMI in kg/m², as well as haemodynamic parameters such as average heart rate (HR) in bpm, mean pulmonary arterial pressure (mPAP) in mmHg, tricuspid regurgitation (TR) volume (TRV) in ml and its fractional volume (f_TRV), shunt fraction (QP/QS), and the corresponding TR severity grade. Furthermore, the antiarrhythmic pharmacotherapy used to manage peri/post-tricuspid valve repair arrhythmias included amiodarone, propranolol, or a dual combination of both, overall symptomatic assessment scales, and a primary focus on the studied outcome of arrhythmia responsiveness.

In this study, we employed two validated symptomatic assessment scales: the four “A” symptomatic (4ASX) score and the New York Heart Association (NYHA) classification I-IV. The NYHA Class I-IV, which emphasises symptoms of left heart failure (HF) such as dyspnoea, is an essential instrument for risk stratification in HF patients. Nonetheless, it has not been explicitly validated for patients with tricuspid regurgitation, which is linked to subtle symptoms resulting from right-heart venous congestion and diminished forward stroke volume. A refined clinical classification emphasising systemic congestion through the manifestations of right heart failure (4A: asthenia, ankle oedema, abdominal discomfort or distension, and anorexia) may possess prognostic significance and assist in identifying patients who might gain from timely intervention.

A binary choice was presented for the arrhythmias at the initial presentation and during subsequent follow-up. Based on antiarrhythmic responsiveness, we primarily categorised the outcomes into two groups: the unresponsive cohort (Cohort I) and the responsive cohort (Cohort II). The study predefined responsiveness to the three categories of antiarrhythmic pharmacotherapies as an average heart rate sustained below 100 bpm, accompanied by a normal electrophysiological electrocardiogram (ECG) during the admission period. The mean pulmonary artery pressure (mPAP) was calculated using the modified Bernoulli equation after applying continuous wave Doppler to the regurgitation jet, ensuring correct alignment. The shunt fraction (QP/QS) was assessed utilising phase contrast Magnetic Resonance Imaginsequences. The severity of TR was evaluated through 2D echocardiography by classifying the fractional TR volume (f_TRV) into four grades. Mild TR is classified for f_TRV below 0.16, moderate TR for f_TRV between 0.16 and 0.25, moderate-severe TR for f_TRV readings between 0.26 and 0.48, and severe TR for f_TRV above 0.48. The f_TRV is determined by dividing the tricuspid regurgitant volume by the right ventricular stroke volume, and the extent of an intra- or extracardiac shunt was assessed by the flow ratio between pulmonary and systemic circulation (Qp/Qs) utilising haemodynamic data.

The previously mentioned tested independent variables were classified according to their estimated average thresholds. In this study, the tested patients were categorised into two groups: older children (≥11 years) and younger children (<11 years up to 2 years). The patients' BMI was categorised into elevated body mass (BMI≥27 kg/m²) and reduced body mass (BMI<27 kg/m2). The patients' average recorded heart rate was categorised into lower heart rate (HR<100 bpm) and higher heart rate (HR≥100 bpm). The decrementing percentage in mean pulmonary arterial pressure was dichotomized into lower decrementing rate (%ΔmPAP<35%) and into higher decrementing rate (%ΔmPAP≥35%), the decrementing rate in tricuspid regurgitation volume was also dichotomized into lower decrementing rate (%ΔTRV<16%) and higher decrementing rate (%ΔTRV≥16%), the decrementing rate in the patients’ shunt ratio was dichotomized into lower decrementing rate (%ΔQP/QS<50%) and higher decrementing rate (%ΔQP/QS≥50%), the decrementing rate in the patients’ fractional tricuspid regurgitant volume was dichotomized into lower decrementing rate (%Δ fTV<12%) and into the higher decrementing rate (%Δ fTV≥12%), the decrementing rate in the New York Heart Association functional classification was dichotomized into lower decrementing rate (%ΔNYHA Class<12%) versus higher decrementing rate (%ΔNYHA Class≥12%), and finally the decrementing rate in the four A symptoms was dichotomized into lower decrementing rate (%Δ4A SXs Score<16%) versus higher decrementing rate (%Δ4A SXs Score≥16%). The decrementing rate was mathematically calculated as the absolute value of the difference between the post-procedural value and the pre-procedural value, divided by the pre-procedural value.

The higher distribution rates in the responsiveness group (Cohort II) for the lower heart rate category (HR<100 bpm) and for the higher decrementing rates categories of the %ΔmPAP≥35%, %ΔTRV≥16%, %ΔQP/QS≥50%, %Δ fTV≥12%, %ΔNYHA Class≥12%, and the %Δ4A SXs Score≥16%, accompanied by statistically significant positive associations as manifested as odd ratio>1, were considered as the outcomes for antiarrhythmics’ efficacy of interest in this study. On the other hand, the safety significance profile for these three adopted antiarrhythmic interventions was identified in this study by the negativity of occurring at least one of the composited side effects of interests (cSE). These cSEs were identified in this study as QT interval prolongation, sinoatrial (SA) or atrioventricular (AV) blocks, alanine/aspartate transaminases (ALT/AST) enzyme elevations, and hyper/hypothyroidism. The higher distribution rates in the responsiveness group (Cohort II) for the negativity of cSE, accompanied by statistically significant positive propensity risk as manifested by odd ratio>1, were considered as the outcomes for antiarrhythmics’ safety of interest in this study.

The previously mentioned independent variables were examined for variations between the two primary comparative groups concerning their responsiveness to the three tested antiarrhythmic categories: amiodarone, propranolol, and dual combinations of amiodarone and propranolol. This analysis was conducted using the chi-square test to illustrate the distribution rates across these two responsiveness groups. Furthermore, the chi-square test expresses the unadjusted risk estimated as an odds ratio along with the p-values. This study utilised Microsoft Excel version 20 (Microsoft Corporation, Redmond, USA) for data collection and filtration, and IBM SPSS Statistics version 25 (IBM Corp., Armonk, USA) for statistical analysis.

## Results

This study examined a total of 300 patients. Approximately 32.33% (97 patients) exhibited clinical unresponsiveness to the study's antiarrhythmic agents, whereas approximately 67.67% (203 patients) attained the desired responsiveness, attaining a stable average heart rate below 100 bpm with normal ECG during admission days. In total, 38% (114 patients) utilised amiodarone as the choice of antiarrhythmic medication. In contrast, 32.3% (97 patients) utilised propranolol as the pharmacotherapeutic intervention, while approximately 29.7% (89 patients) experienced the dual combination of amiodarone and propranolol. However, we didn’t show statistically significant variations in the conducted antiarrhythmic pharmacotherapies across the two based responsiveness tested cohorts (p-value = 0.594).

A total of 172 females (57.3%) and 128 males (42.7%) were tested, with no statistically significant differences observed between the responsiveness cohorts (p-value = 0.274). Approximately 50.3% (151 patients) were under 11 years of age, while 49.7% (149 patients) were over 11 years of age. However, there was statistically insignificant variation across Cohort I-II in this study (p-value = 0.053).

Of importance, we showed in this study a statistically significant variation in the occurrence of major side effects of the tested antiarrhythmic agents with a higher distribution rate in the responsiveness group compared to the unresponsiveness group [88 (43.3%) vs. 13 (13.4%)], respectively, for the negativity of cSE. In contrast, the responsiveness group showed lower distribution rates compared to the unresponsiveness group [115 (56.7%) vs. 84 (86.6%), respectively] regarding the positivity category of cSE (occurring at least one of the major side effects that encompasses, as predefined, SA/AV block, haemodynamic instability, alanine transaminase (ALT) to aspartate transaminase (AST), elevation, and hypo/hyperthyroidism circumstances).

Anthropometrically, we didn’t show statistically significant variations across responsiveness-related cohorts in this study regarding distributional rates for higher versus lower body mass indexes (BMI≥27 kg/m2 vs. BMI<27 kg/m2) with a p-value of 0.115. Our study revealed an overall higher body mass rate of 162 (54.0%) compared to a lower body mass rate of 138 (46.0%). The distribution rates for patients’ higher body masses in the responsiveness cohort were insignificantly higher than in the unresponsiveness cohort [114 (56.2%) vs. 48 (49.5%), respectively]. We identified statistically significant distribution rates concerning patients' heart rates, <100 bpm versus ≥100 bpm, across Cohorts I-II (p-value = 0.003), where the responsive cohort comprised approximately 56.7% (115 patients) in contrast to approximately 38.1% (37 patients) in the unresponsive cohort among the lower heart rate (HR <100 bpm) compared to a lower distributional rate of 88 (43.3%) in the responsiveness cohort compared to 60 (61.9%) in the unresponsiveness cohort among patients with ≥100 bpm category.

This study showed statistically significant variations across responsiveness-related treated cohorts (Cohort I and Cohort II) among haemodynamically tested variables %ΔTRV (adopted 16% as a dichotomized threshold) and %Δ fTV (adopted 12% as a dichotomized threshold), while we didn’t show statistically significant variation across Cohort I-II among haemodynamically tested variables of %ΔmPAP (adopted 35% as a dichotomized threshold) and %ΔQP/QS (adopted 50% as a dichotomized threshold). The distributional rates for these haemodynamically tested variables among their higher categories (≥adopted thresholds) in the responsiveness cohort were expressed at [82 (40.4%), 110 (54.2%), 98 (48.3%), and 99 (48.8%), respectively] compared to those in the unresponsiveness cohort [15 (15.5%), 23 (23.7%), 36 (37.1%), and 41 (42.3%), respectively].

Symptomatically, this study showed statistically significant variation in the more specified symptoms score, the 4ASx score, but not in the non-specified symptomatic assessing score, the NYHA Class I-IV scoring system. This study revealed a higher distributional rate for the higher decrementing rate categories (%Δ4A SXs Score≥16% and %ΔNYHA Class≥12%) in the responsiveness cohort (Cohort II) compared to that in the unresponsiveness cohort (Cohort I) [115 (56.7%) and 135 (66.5%), versus [38 (39.2%) and 62 (63.9%), respectively].

**Figure 1:**
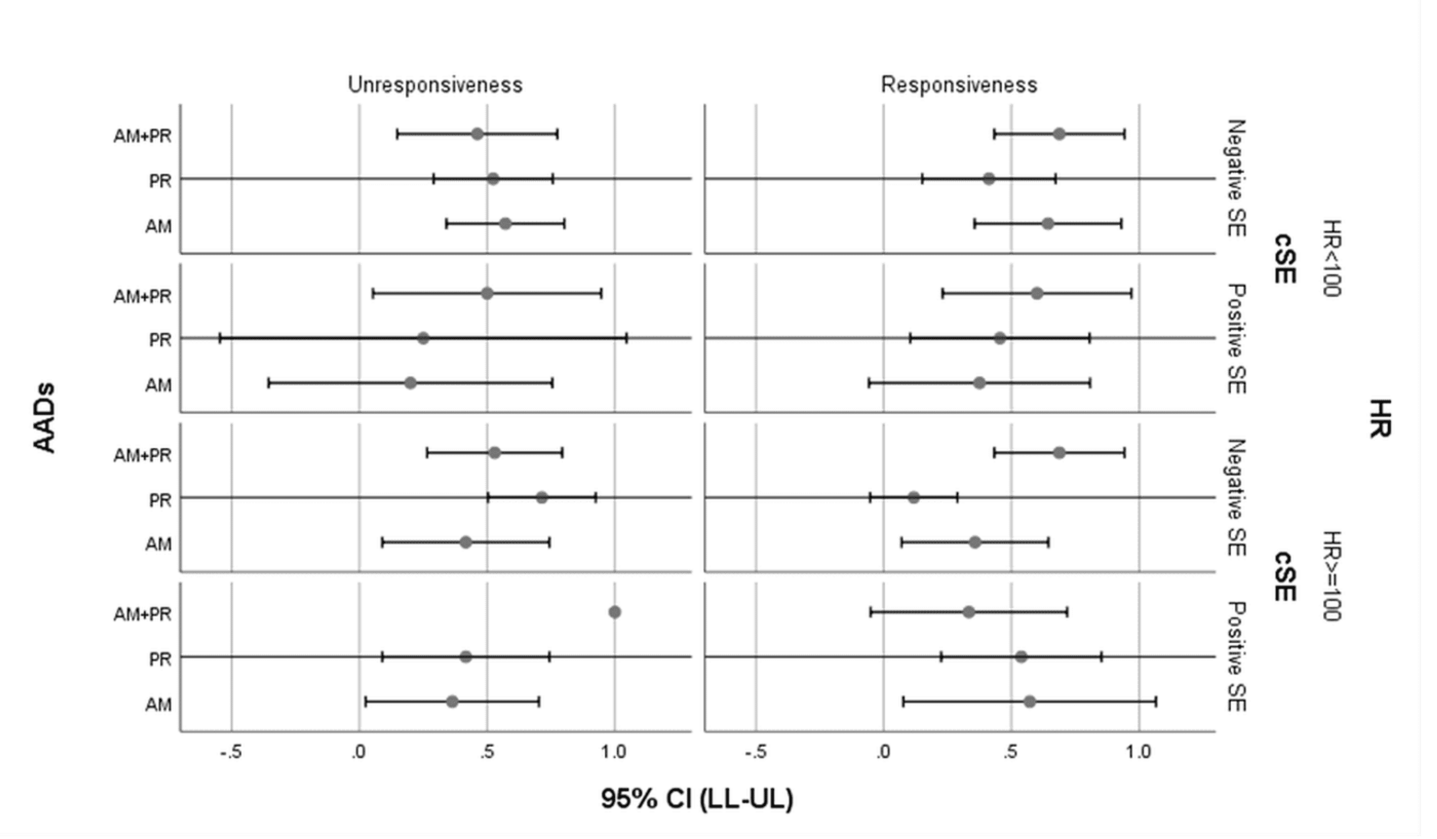
Illustration of an error bar chart for multi-level comparisons of patients' heart rate and cSE variables across responsive groups utilising AADs. Error bar representations for the patients' efficacy-related variable, the HR, and the patients' safety-related tested variable, the cSE, across the patients' utilized AAD of investigations responsiveness. Responsiveness in this study was predefined as the persistence of average HR<100 beats/min with normal ECG rhythms during admission. The cSE profile in this study encompassed the evidence of SA and AV nodal blocking, hemodynamic instability, and hypo/hyperthyroidism episodes. AAD of investigation in this study encountered either amiodarone, propranolol, or a combination of both. HR: Heart rate; cSE: Coposited side effect; AAD: Antiarrhythmic drug; CI: Confidence interval; LL: Lower limit; UL: Upper limit; AM: Amiodarone; PR: Propranolol; SA: sinoatrial; AV: atrioventricular; SE: side effect.

The results of comparative analyses for paediatric demographic, haemodynamic, and symptomatic variables across the two responsiveness groups to the three investigated antiarrhythmic drugs are shown in below Table 1. An illustration of an error bar chart for multi-level comparisons of patients' heart rate and cSE variables across responsive groups utilising AADs is presented in Figure 1.

**Table 1:** Results of comparative analyses for paediatric demographic, haemodynamic, and symptomatic variables across the two responsiveness groups to the three investigated antiarrhythmic drugs. A chi-square test was performed to analyse paediatric demographic, haemodynamic, and symptomatic variables between two responsiveness groups: the unresponsive group (Cohort I) and the responsive group (Cohort II), concerning three intravenous antiarrhythmic drugs: amiodarone, propranolol, and their dual combination, during admission following transcatheter intervention for congenital heart disease affecting the tricuspid valve in paediatric patients. The outcomes were presented as distributional rates and propensity risk ratios. These cSEs were identified in this study as QT interval prolongation, sinoatrial (SA) or atrioventricular (AV) blocks, alanine/aspartate transaminases (ALT/AST) enzyme elevations, and hyper/hypothyroidism. AAD: Antiarrhythmic drug; cSE: Composited side effects for the; BMI: Body mass index; HR: Heart rate; %ΔmPAP: Decrementing rate in mean pulmonary artery pressure; %ΔQP/QS: Decrementing rate in shunt ratio; %ΔTRV: Decrementing rate in tricuspid regurgitant volume; %Δ fTV: Decrementing rate in fractional tricuspid regurgitant volume; %Δ4A SXs Score: Decrementing rate patients four “A” symptoms (asthenia, ankle oedema, abdominal discomfort or distension, and anorexia); %ΔNYHA: Decrementing rate in New York Heart Association classification.

	AAD Unresponsiveness (Cohort I) (97, 32.33%)	AAD Responsiveness (Cohort II) (203,67.67%)	Overall 300	Odd Ratio	p-value
Gender					
Female	60 (61.9%)	112 (55.2%)	172 (57.3%)	1.318 (95%;0.804-2.160)	0.274
Male	37 (38.1%)	91 (44.8%)	128 (42.7%)		
Age (yrs)					
<11	41 (42.3%)	110 (54.2%)	151 (50.3%)	0.619 (95% CI; 0.380-1.009)	0.053
≥11	56 (57.7%)	93 (45.8%)	149 (49.7%)		
cSE					
Positive	84 (86.6%)	115 (56.7%)	199 (66.3%)	4.944 (95% CI; 0.590-9.441)	0.000
Negative	13 (13.4%)	88 (43.3%)	101 (33.7%)		
BMI (Kg/m2)					
<27	49 (50.5%)	89 (43.8%)	138 (46.0%)	1.308 (95% CI; 0.805-2.124)	0.115
≥27	48 (49.5%)	114 (56.2%)	162 (54.0%)		
HR (bpm)					
≥100	60 (61.9%)	88 (43.3%)	148 (49.3%)	2.119 (95% CI; 1.292-3.476)	0.003
<100	37 (38.1%)	115 (56.7) %	152 (50.7%)		
%ΔmPAP					
<35%	61 (62.9%)	105 (51.7%)	166 (55.3%)	1.581 (95% CI; 0.964-2.596)	0.069
≥35%	36 (37.1%)	98 (48.3%)	134 (44.7%)		
%ΔQP/QS					
<50%	56 (57.7%)	104 (51.2%)	160 (53.3%)	1.300 (95% CI; 0.798-2.118)	0.291
≥50%	41(42.3%)	99 (48.8%)	140 (46.7%)		
%ΔTRV					
<16%	82 (84.5%)	121 (59.6% )	203 (67.7%)	3.705 (95% CI; 1.998-6.871)	0.000
≥16%	15 (15.5% )	82 (40.4% )	97 (32.3%)		
%Δ fTV					
<12%	74 (76.3%)	93 (45.8%)	167 (55.7%)	3.806 (95% CI; 2.210-6.552)	0.000
≥12%	23 (23.7%)	110 (54.2%)	133 (44.3%)		
AAD					
Amiodarone	39 (40.2%)	75 (36.9%)	114 (38.0%)	NA	0.594
Propranolol	33 (34.0%)	64 (31.5%)	97 (32.3%)		
Combination	25 (25.8%)	64 (31.5%)	89 (29.7%)		
%Δ4A SXs Score					
<16%	59 (60.8%)	88 (43.3%)	147 (49.0%)	2.029 (95% CI;1.239-3.323)	0.005
≥16%	38 (39.2%)	115 (56.7%)	153 (51.0%)		
%ΔNYHA Class					
<12%	35 (36.1%)	68 (33.5%)	103 (34.3%)	1.121 (95% CI; 0.675-1.860)	0.058
≥12%	62 (63.9%)	135 (66.5%)	197 (65.7%)		

## Discussion

Antiarrhythmic drugs are crucial in treating heart rhythm disturbances, which can be physiological or cause serious issues. These drugs can cause significant harm to organs and systems and are considered one of the main causes of sudden death. The evolution of antiarrhythmic agents has been a protracted and intricate endeavour, commencing with the initial medical application aimed at enhancing blood clot formation in irregular arrhythmias through the utilisation of a particular plant [12]. Antiarrhythmic therapy is effective, but it is advisable to prescribe combinations of drugs for severe forms of arrhythmias [13]. Amiodarone and propranolol have different mechanisms of action, with amiodarone increasing cardiac action potential by blocking sodium, calcium, and potassium channels, and propranolol decreasing heart rate and oxygen consumption. These drugs play important roles in their clinical prognosis and outcomes. Amiodarone is used for atrial and ventricular arrhythmias in heart failure and coronary spasm patients, while propranolol is used for atypical chest pain and hypertension. The oral bioavailability of amiodarone is low, while propranolol is nearly 100% [14].

Antiarrhythmic medications are crucial in treating patients with arrhythmic disorders, such as atrial fibrillation and ventricular arrhythmias in ischemic heart disease. Clinical studies have shown that amiodarone can treat almost all types of arrhythmias with a proven broad spectrum of efficacy. Propranolol also seems effective in treating tachyarrhythmias. Knowledge of the drug's various side effects is crucial for proper management in patients with various comorbidities [15]. Amiodarone is effective in treating all forms of arrhythmias, including ventricular tachycardia, atrial fibrillation, and ventricular fibrillation. It is suitable for pregnant and young patients due to its low adverse events. Numerous studies have shown that propranolol and amiodarone are both effective for treating tachyarrhythmias, with advantages such as the most rapid action of propranolol in re-establishing the sinus rhythm and the low rate of recurrence of arrhythmias with amiodarone treatment [16]

However, based on the currently available clinical studies, it is difficult to determine if amiodarone is superior or inferior to propranolol. Multiple variables can influence the responsiveness of each patient, such as serum drug levels within the therapeutic range, genetic variability in amiodarone or propranolol pharmacokinetics or pharmacodynamics, drug interactions, pharmacogenomics, concentration of metabolites, individuals' lifestyles, and follow-up monitoring [17]. Some studies have shown that amiodarone is more beneficial than propranolol for patients with underlying structural heart disease, particularly in the elderly and those with atrial fibrillation or class IV indications. However, these clinical studies do not provide enough evidence to be applied to populations of other ages, causative factors for amiodarone or propranolol use, comorbidities, and situations where there is no follow-up monitoring by physicians weekly and ultrasound information at one month [18].

A study examined predictors of treatment with amiodarone, propranolol, or sotalol in arrhythmic patients, finding a positive relationship between the use of beta-blockers and the use of amiodarone and treatment acceptance. This suggests that patients who had the opportunity to use amiodarone and refused the propranolol proposal were more likely to refuse amiodarone treatment unconditionally [19]. In conclusion, it remains difficult to compare the effectiveness and safety of medications that are not exactly substitutable in a comparative effectiveness approach. Future trials focusing on innovations in nondrug therapies and novel antiarrhythmic medications could open new fields for improving antiarrhythmic therapy. Education not only for cardiologists but also for all physicians in contact with patients with atrial fibrillation is a priority, as the selection of a new antiarrhythmic drug has become an increasingly complex choice [20].

Akin A et al. conducted a study on the safety and effectiveness of amiodarone and propranolol combination therapy for infant arrhythmias. The study involved 2-18-year-olds and found a 92% success rate. Twenty patients had complete arrhythmia control within two months, while three had short attacks. The study suggests this treatment may lead to ablation therapy in older children [21]. A meta-analysis by Ardaya R et al. compared amiodarone and beta-blockers in preventing postoperative atrial fibrillation (AF) after cardiac surgery. The study found no significant difference in AF episodes, duration, hospital stay, or mean ventricular rate between the two groups, concluding that both medications effectively prevent AF [22]. Barton et al.'s study on infant supraventricular arrhythmias found that high-dose propranolol monotherapy effectively treats the condition. The therapy, which began at 17 days, controlled SA in 67.3% of patients. However, one patient required discontinuation due to adverse events. The study suggests that inpatient control may predict outpatient efficacy [23].

Indeed, the study by Solomon AJ, et al. revealed that early intravenous amiodarone, followed by oral amiodarone, may be superior to propranolol in preventing postoperative atrial fibrillation. It is well-accepted and can be initiated at the time of the surgical procedure. Nonetheless, the administration of amiodarone did not lead to a decrease in the duration of hospitalisation. Furthermore, the study by Solomon AJ, et al. demonstrated that amiodarone is superior to propranolol in decreasing the incidence of atrial fibrillation. This incidence was diminished by approximately 50%. Notwithstanding this decrease in atrial fibrillation, amiodarone did not diminish the duration of hospitalisation. This was likely a consequence of the prolonged duration of atrial fibrillation in patients treated with amiodarone [24].

Despite being statistically insignificant in this study, the elevated prevalence of male paediatric subjects in the responsiveness group, cohort II (91 (44.8%), compared to the unresponsiveness group, cohort I (37 (38.1%), along with the positive propensity for responsiveness in males versus females 1.318 (95%; 0.804-2.160), may hold clinical relevance for this affected paediatric cohort within the overall population. Furthermore, although the variation between Cohort I and II concerning paediatric patients tested and categorised into binary groups using an 11-year threshold was statistically insignificant, we demonstrated a diminished propensity for responsiveness, which may hold clinical significance, to antiarrhythmic pharmacotherapies as patients affected by TV age (0.619 (95% CI; 0.380-1.009)].

This study demonstrated an overall occurrence rate of at least one of the monitored major side effects - SA/AV block, haemodynamic instability, AST/ALT elevation, and hypo/hyperthyroidism - at 199 (66.3%), with an overall negativity rate for cSE incidence of 101 (33.7%). The propensity for negative cSE incidence compared to positive cSE incidence was quantified at 4.944 (95% CI; 0.590-9.441) when the examined antiarrhythmic medications demonstrated clinical efficacy in stabilising heart rate and normalising the ECG of admitted paediatric patients during hospitalisation. Despite being statistically insignificant in this study concerning the potential effects of paediatric patients' body mass indices, we demonstrated a greater likelihood of responsiveness to antiarrhythmic intravenous drugs associated with higher body masses (1.308 (95% CI; 0.805-2.124)).

This study indicates that approximately 50.7% (152 patients) exhibited an average heart rate below 100 bpm with a normal ECG pattern upon admission, while approximately 49.3% (148 patients) had an average heart rate exceeding 100 bpm with unstable ECG readings during admission. The propensity ratio for utilising the average heart rate threshold of 100 bpm as a distinguishing criterion for higher versus lower categories was examined at 2.119 (95% CI; 1.292-3.476).

Our study demonstrated elevated distribution rates for the categories with higher decrementing rates concerning the assessed haemodynamic variables of %ΔmPAP, %ΔQP/QS, %ΔTRV, and %ΔfTV. Although these findings were statistically insignificant, they may hold clinical significance for %ΔmPAP and %ΔQP/QS in the responsiveness cohort (Cohort II) compared to the unresponsiveness cohort (Cohort I). This suggests favourable haemodynamic outcomes relevant to the administration of antiarrhythmic drugs during the peri/post-operative period of tricuspid valve repair in paediatric patients with congenital atrial septal defects. The propensity ratios for the normalisation of patients' heart rates and ECG patterns were 1.581 (95% CI; 0.964-2.596), 1.300 (95% CI; 0.798-2.118), 3.705 (95% CI; 1.998-6.871), and 3.806 (95% CI; 2.210-6.552), respectively, when the aforementioned haemodynamically tested variables surpassed their estimated thresholds of 35%, 50%, 16%, and 12%, respectively.

This study also revealed a positive correlation between the greater decrement rates in the adopted symptomatic assessment scores, 4ASx and NYHA I-IV scoring systems, despite statistical insignificance in the NYHA I-IV. However, this correlation may hold clinical significance for the small cohort of paediatric patients with congenital conditions regarding their responsiveness to antiarrhythmic medications. The propensity ratios for normalising patients' heart rates and ECG patterns were found to be 2.029 (95% CI; 1.239-3.323) and 1.121 (95% CI; 0.675-1.860), respectively, when these tested variables surpassed their estimated thresholds of 16% and 12%, respectively.

The decision-making process in managing paediatric arrhythmias frequently lacks transparency. The findings indicate that either intravenous propranolol or amiodarone, or their combinations, may be utilised effectively and safely in paediatric patients with congenital heart defects, particularly atrial septal defects, serving as a representative example for other congenital cardiac conditions.

The retrospective design included only paediatric patients undergoing TV repair; the single-centre approach and relatively small sample size, which are likely constrained the study's external validity and generalizability, were considered a limitation for this study. Larger, controlled studies encompassing a broader range of congenital heart disease surgical procedures, including multi-centre international research, are necessary.

## Conclusions

The use of antiarrhythmic pharmacotherapies during the perioperative or postoperative period of tricuspid valve repair was statistically significant in terms of achieving responsiveness statuses against procedural-related arrhythmia. This was accompanied by a positive outcome in corresponding symptomatic and haemodynamic monitored parameters, and the safety profile was statistically significant and non-inferior. This was the case regardless of the antiarrhythmic agents that were utilised, which could be amiodarone, propranolol, or a combination of the two. Our research indicates that therapeutic protocols for paediatric patients necessitating antiarrhythmic intervention post-cardiac surgery should undergo substantial revision.
